# Photosystem II Photochemistry and Phycobiliprotein of the Red Algae *Kappaphycus alvarezii* and Their Implications for Light Adaptation

**DOI:** 10.1155/2013/256549

**Published:** 2013-11-27

**Authors:** Xiangyu Guan, Jinfeng Wang, Jianyi Zhu, Chunyan Yao, Jianguo Liu, Song Qin, Peng Jiang

**Affiliations:** ^1^School of Ocean Sciences, China University of Geosciences, Beijing 100083, China; ^2^Key Laboratory of Experimental Marine Biology, Institute of Oceanology, Chinese Academy of Sciences, Qingdao 266071, China; ^3^Department of Biology, Changshu Institute of Technology, Changshu 215500, China; ^4^Yantai Institute of Coastal Zone Research, Chinese Academy of Sciences, Yantai 264003, China

## Abstract

Photosystem II photochemistry and phycobiliprotein (PBP) genes of red algae *Kappaphycus alvarezii*, raw material of **κ**-carrageenan used in food and pharmaceutical industries, were analyzed in this study. Minimum saturating irradiance (*I*_*k*_) of this algal species was less than 115 **μ**mol m^−2^ s^−1^. Its actual PSII efficiency (yield II) increased when light intensity enhanced and decreased when light intensity reached 200 **μ**mol m^−2^ s^−1^. Under dim light, yield II declined at first and then increased on the fourth day. Under high light, yield II retained a stable value. These results indicate that *K. alvarezii* is a low-light-adapted species but possesses regulative mechanisms in response to both excessive and deficient light. Based on the PBP gene sequences, *K. alvarezii*, together with other red algae, assembled faster and showed a closer relationship with LL-*Prochlorococcus* compared to HL-*Prochlorococcus*. Many amino acid loci in PBP sequences of *K. alvarezii* were conserved with those of LL-*Prochlorococcus*. However, loci conserved with HL-*Prochlorococcus* but divergent with LL-*Prochlorococcus* were also found. The diversities of PE and PC are proposed to have played some roles during the algal evolution and divergence of light adaption.

## 1. Introduction

*Kappaphycus alvarezii* (Doty) Doty (Rhodophyta, Solieriaceae) is a commercially important marine alga for its high production of polysaccharide *κ*-carrageenan, which is commonly used in food, medicine, and cosmetic industries [1, 2]. Extensive application and increasing demand make* K. alvarezii* widely cultivated around Southeast Asia, East Africa, and South America and promote approximately 8% growth rates annually in the carrageenan industry [3, 4]. Photosynthetic responses of *K. alvarezii* have frequently been detected to clarify the optimal conditions that would maximize its photosynthesis and growth. For example, the effects of temperature, salinity, and UVB radiation on different morphotypes of *K. alvarezii* were evaluated under controlled conditions [5–7]. However, little is known about their physiological changes associated with light intensities, and the optimal photosynthetic available radiation levels of many locally cultivated strains remain to be determined. Photosystem (PS) II photochemical parameters measured by pulse amplitude modulation (PAM) fluorometry have long been used for evaluating the photosynthetic physiology of land plants [8, 9]. Moreover, its application development can easily be found in algal research [10–12]. It should be a feasible tool for *in vivo* testing of the photosynthetic behaviors of *K. alvarezii* to different radiations as well as for ascertaining the best light conditions.

Photosynthetic behaviors and light response of plants are largely determined and regulated by their photosynthetic units [13, 14]. Phycobilisomes (PBSs) are major photosynthetic units that endue* K. alvarezii* and other red algae, cyanobacteria, several cryptomonads, and brown algae with the capability of light-harvesting and energy migration [15]. For their biomedical value, combinational biosynthesis and purification of recombinant PBSs have been carried out with the help of modern biotechnology [16, 17]. PBSs are composed of linker polypeptides and phycobiliproteins (PBPs), mainly allophycocyanin (APC), phycoerythrin (PE), and phycocyanin (PC). PBSs are one of the ways by which algae survive in varying and sometimes extreme habitats [18]; some examples would be *Prochlorococcus* and *Synechococcus*, two genera of abundant unicellular organisms. *Prochlorococcus* consist of two ecotypes specifically adapted to either low-light (LL) or high-light (HL) conditions. Their pigmentation and gene complement are quite different [19]. By altering their PBPs structure and PBSs composition, such as the PE divergence in LL-*Prochlorococcus *and HL-*Prochlorococcus* [20, 21], algae evolve to accommodate different light conditions. Prior studies demonstrated that PE genes in LL-*Prochlorococcus* suffer from positive selection, and the selected sites are related to light-harvesting or energy-transferring, which is consistent with the findings in other PBPs [22]. Similarly, based on their PBS structure and composition,* Synechococcus* were divided into three types: type 1, rods are composed of C-PC only; type 2, rods are composed of either C-PC or R-PCIII and a PEI-like PBP; type 3, rods are composed of R-PC and two PE types (PEI and PEII) [23]. Accordingly, nucleic or amino acid sequences of PBPs and PBSs linker family may have hidden some helpful information related to algal photosynthesis and light adaptation [24, 25]. Unfortunately, so far, only PBP genes of very few red algae such as *Aglaothamnion neglectum* and *Gracilaria tenuistipitata* were sequenced [26, 27]. The information of *K. alvarezii* is still unknown.

The present study measured PSII photochemistry to evaluate photosynthetic efficiency and light response of *K. alvarezii*. In addition, PBP genes were firstly sequenced and aligned with those of other algae. The results of this study would improve our understanding of the impact of changing light conditions on *K. alvarezii* and the potential mechanism of light adaption of this red alga.

## 2. Materials and Methods

### 2.1. Algal Material

*K. alvarezii* were collected from a farm population in Lingshui, Hainan Province, China. Healthy fronds were cultivated in N/P-enriched seawater (N, 0.43 mM; P, 0.019 mM) at 23°C under 12 h : 12 h (L:D) photoperiod and 40 *μ*mol m^−2^ s^−1^ light intensity. The culture medium was renewed once a week.

### 2.2. Chlorophyll Fluorescent Measurement

Chlorophyll fluorescence of *K. alvarezii* thalli was cultivated under a light intensity of 40 *μ*mol m^−2^ s^−1^ and then cultivated under five different light intensities of 10, 25, 50, 100, and 200 *μ*mol m^−2^ s^−1^ in four days. Healthy samples were dark-adapted for 15 min; after which actual PSII efficiency (yield II) and rapid light curves (RLC) were measured using a Water-PAM chlorophyll fluorometer with Water-EDF Fiberoptics-Emitter-Detector Unit (Walz, Effeltrich, Germany). The steady-state fluorescence level during exposure to cultivated light intensity (Ft) was detected by measuring the value under a 0.3 *μ*mol m^−2^ s^−1^ modulated light and a 40 *μ*mol m^−2^ s^−1^ actinic light. Maximum fluorescence level during illumination (Fm′) was measured by a 0.8 s saturating pulse at 4000 *μ*mol m^−2^ s^−1^. Yield II was calculated according to the formula yield II = (Fm′ − Ft)/Fm′. Ft and Fm′ were measured under nine different and increasingly actinic lights (PAR = 0, 43, 64, 92, 138, 209, 309, 479, and 714 *μ*mol m^−2^ s^−1^) of a 10 s duration and a 0.8 s saturating pulse at 4000 *μ*mol m^−2^ s^−1^. Relative electron transport rate (rETR) was calculated as rETR = yield × PAR ×  *A*  × 0.5, in which PAR symbolizes responses to photosynthetic active radiation (*A*, absorbancy index = 0.85 in this study). From the RLC, relative maximum electron transport rate (rETRmax) and initial slope (*α*) were obtained. Minimum saturating irradiance (*I*_*k*_) = rETRmax/*α*. Results were expressed as mean ± SD, in which data from three independent experiments were analyzed with the statistical software STATISTICA7.0 (*t*-test, *P* < 0.05).

### 2.3. DNA Extraction and Sequencing of PBP Genes

Algal samples were ground in liquid nitrogen and the genomic DNA was extracted with a Plant Genome DNA Kit (Tiangen, Beijing, China) according to the manufacturer's instructions.

Based on the conserved sequences of red algae *A. neglectum* (Z11906, Z11905), *Ceramium boydenii* (AF526383), *Gracilaria lemaneiformis* (AF275685), *G. tenuistipitata* var. *liui* (AY673996), *Griffithsia monilis* (Z98528), *Porphyra haitanensis* (DQ449071, AY372218), and *Porphyra yezoensis* (DQ666487), degenerate oligonucleotide primers of PBP genes were designed (Table 1). The PCR program utilized an initial denaturation at 94°C for 5 min, followed by 35 cycles at 94°C for 1 min, 47°C for 1 min, and 72°C for 1 min 30 s, with a final elongation at 72°C for 10 min. Amplification reactions were performed in a Biometra Thermal Cycler (Biometra, Gottingen, Germany).

PCR products were purified with a TIANgel Midi Purification Kit (Tiangen, Beijing, China) and then constructed into a pMD18-T vector system (Takara, Dalian, China). The ligation products were transferred into *Escherichia coli *TOP 10 strains. Five positive clones of each amplification product were checked by their electrophoretic mobility and PCR analysis after the recombinant plasmids were selected under a 100 *μ*g mL^−1^ concentration of ampicillin. Double strands of these target segments were sequenced by Shanghai Sunny Biotechnology Co., Ltd. (Sunny, Shanghai, China).

### 2.4. Multiple Sequence Alignment and Phylogenetic Analysis

A twenty-four-cyanobacterial-genome database was accessed from JGI (http://www.jgi.doe.gov/) in FASTA format, and the species *Gloeobacter violaceus* (GV),* Trichodesmium erythraeum* (TE),* Synechococcus* (S.), and* Prochlorococcus* (P.) were examined. PBP sequences of Rhodophyta (including species of Bangiophyceae and Florideophycease), *Cyanophora paradoxa* (M11159, CP), and *Guillardia theta *(AM183803) were searched at NCBI [28, 29]. PBP nucleotide sequences of *K. alvarezii* (KA) were cloned in this paper and translated to amino acid sequences by Omiga [30]. Protein sequences of the PBSs previously described were used as database queries. Each protein in this query dataset was used to search for potential novel sequences in above sequenced cyanobacterial species genomes using the BLASTP and TBLASTN programs [31]. Sequences giving better reciprocal BLAST hits were assumed to be capable of identifying homologous counterparts in these species if they could be aligned with at least the BLAST-Score >90 and the *E*-value <1*e* ^−10^. The search was iterated until convergence and then examined individually.

Multiple protein sequences alignment was performed using ClustalX [32]. Neighbor joining (NJ) and maximum parsimony (MP) methods in MEGA5 were used to construct the phylogenetic tree [33], in which the confidence level of each branch was determined by analyzing 1000 bootstrap replicates. Bootstrap values >50% were generated.

### 2.5. Tertiary Structure Prediction

Tertiary structure of PE *α*- and *β*-subunits of *K. alvarezii* was analyzed using homology modeling. Next, *α*- and *β*-PE amino acid sequences of *K. alvarezii* were submitted to the protein-modeling server SWISSMODEL (http://swissmodel.expasy.org/) and predicted with PDB-1b8dK using PDB-1liaB as the model template. All the manipulations were performed using PdbViewer.

## 3. Results

### 3.1. PSII Photochemical Efficiency

**Figure 1:**
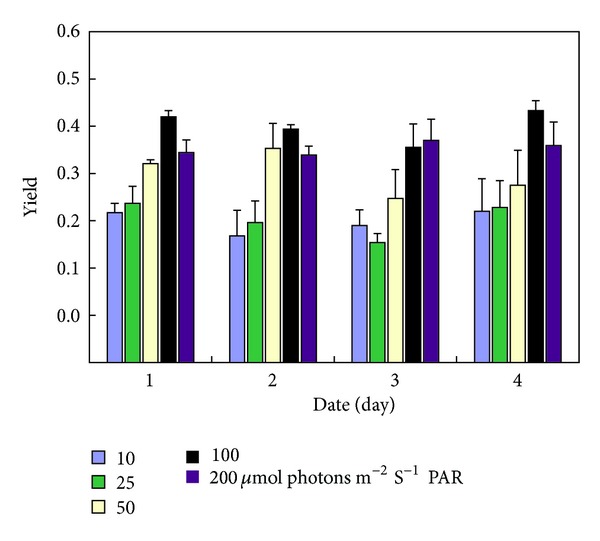
Variation of actual PSII efficiency (yield II) of *Kappaphycus alvarezii* grown under different light intensities. Indicated data are the mean data of three independent experiments (±SD).

PSII photochemical parameters of *K. alvarezii* thalli are shown in Table 2. The value *I*_*k*_ is less than 115 *μ*mol m^−2^ s^−1^ for *K. alvarezii*. Yield II increased when cultivated light intensity enhanced from 10 *μ*mol m^−2^ s^−1^ to 100 *μ*mol m^−2^ s^−1^ and decreased when the light intensity reached 200 *μ*mol m^−2^ s^−1^. Yield II declined during 1 d~3 d under dim light (less than 100 *μ*mol m^−2^ s^−1^) at first and then rose on the 4th day. Under high light (200 *μ*mol m^−2^ s^−1^), yield II retained a stable value, with a little higher value on the 3rd day (Figure 1).

**Figure 2:**
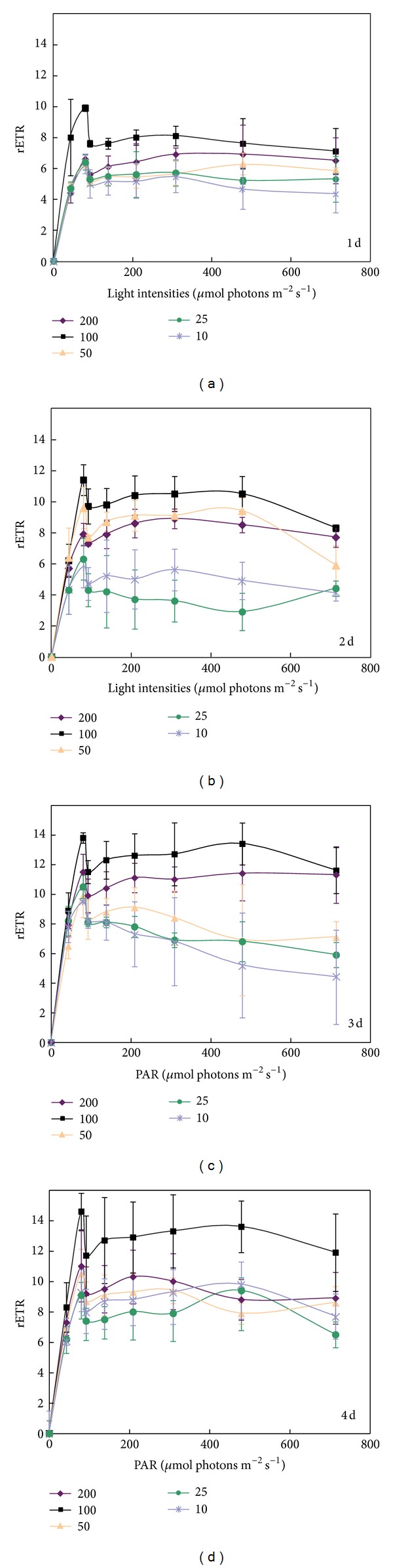
Rapid light curves (RLCs) of *Kappaphycus alvarezii* cultivated under different light intensities. Indicated data are the mean data of three independent experiments (±SD).

The RLCs of *K. alvarezii* cultivated under different light intensities during four days are shown in Figure 2. Under the condition of low PAR, all rETRs rose rapidly; rETRs reached their maximum when actinic light was approximately 80 *μ*mol m^−2^ s^−1^, declined significantly as soon as PAR exceeded 80 *μ*mol m^−2^ s^−1^, and then climbed slightly. The rETRs were lower under dim light than under high light. It was highest under a cultivated light intensity of 100 *μ*mol m^−2^ s^−1^. Regardless of the light intensity (dim or high), rETRs reached a relatively high value from the first day to the fourth day.

### 3.2. Phylogenetic Relationship Inferred by PE Sequences

**Figure 3:**
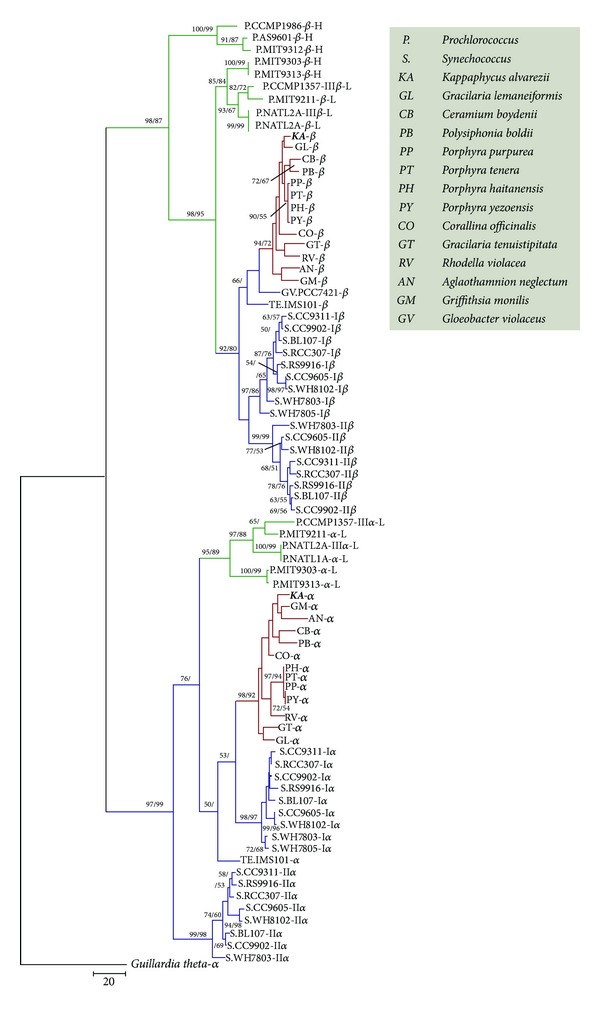
Phylogenetic tree of *Kappaphycus alvarezii*, 12 other red algae, and sequenced cyanobacteria based on PE. Numbers at nodes indicate branch support given as bootstrap values from neighbor joining (NJ)/maximum parsimony (MP) analysis. Numbers are only shown if they exceed 50, respectively.

The relationship among PE sequences of *K. alvarezii*, 12 other red algae, and 22 completed sequenced cyanobacteria was investigated. With *α*-PE of *G. theta* as the root, an alignment of 78 identified *α*- and *β*-subunits amino acid sequences of PE was followed by the generation of an NJ and MP phylogenetic tree (Figure 3). The *α*-subunit PE (including *α*-subunit PE of red algae, four clusters *α*-subunit PEI of* Synechococcus*, PEII of* Synechococcus*, and *α*-subunit PEIII of *Prochlorococcus*) and *β*-subunit PE (including *β*-subunit PE of red algae, three clusters PEIII of HL-*Prochlorococcus*, PEIII of LL-*Prochlorococcus*, and PEI and PEII of* Synechococcus*) were distinctly assembled into two monophyletic groups. In the *β*-subunit group, red algae clustered first with the two cyanobacteria *G. violaceus* and *T. erythraeum* and then assembled with the PEI and PEII clades of* Synechococcus*. Compared with HL-*Prochlorococcus*, they have a closer relationship with LL-*Prochlorococcus*. Red algal PE sequences are more similar to LL-*Prochlorococcus* PEs than to HL-PEs. A possible explanation of this result is that HL-*Prochlorococcus* evolves faster than LL, and also exhibits much higher GC content. Hence, the similarities among red algal-, HL-, and LL-PEs do not necessarily reflect their photosynthetic properties and relationships. In the *α*-subunit group, red algae successively assembled with PEI of* Synechococcus*, LL-*Prochlorococcus*, and *T. erythraeum*; PEII of* Synechococcus* formed a cluster by itself.

### 3.3. Tertiary Structure of PE

**Figure 4:**
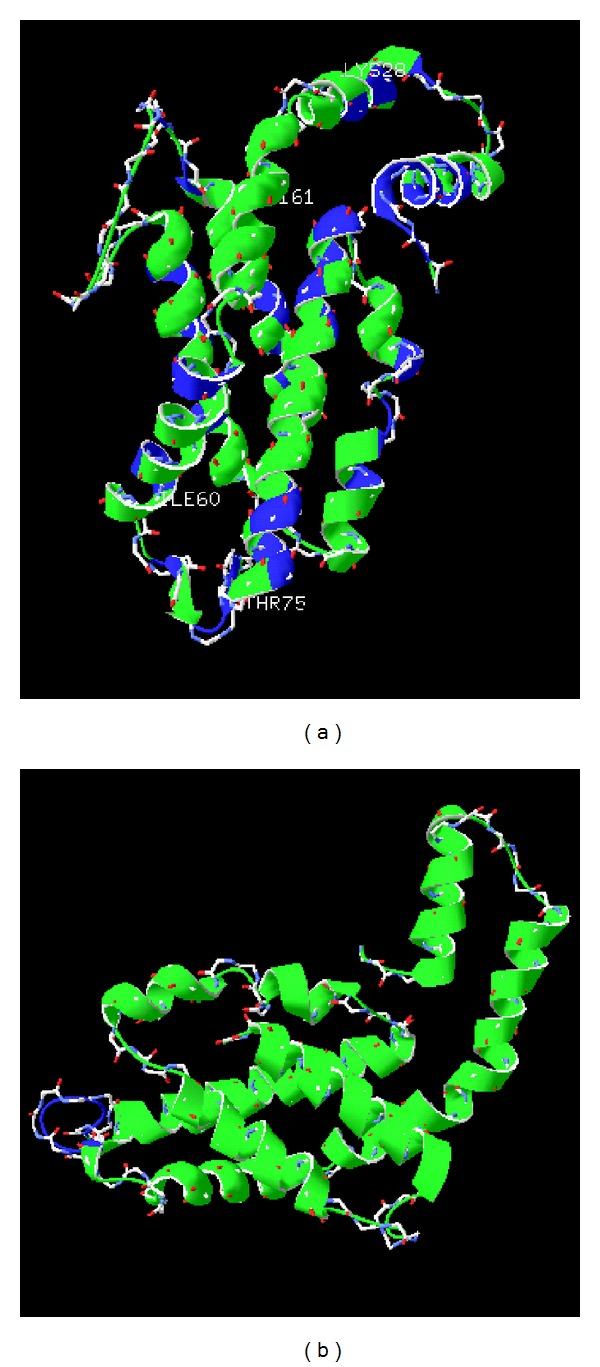
Predicted structure model of PE *β*-subunit (a) and *α*-subunit (b) of *Kappaphycus alvarezii*. PDB-1b8dK and PDB-1liaB were chosen as the model templates, respectively.

Forty-eight amino acid loci conserved with LL-*Prochlorococcus* but diverged with HL-*Prochlorococcus* were mapped onto *β*-PE tertiary structure of *K. alvarezii* in blue color (Figure 4(a)). These loci distribute to the following six domains: (1) N-terminal 2-10aa domain is obviously conserved, including the loci 3D function of interaction of N-terminus of *α*- and *β*-subunits; (2) 65-81aa domain (except 67I, 68A, 72N, 75T, 76N, and 79M) is adjacent to the motif of chromophore interaction; (3) 92-100aa domain (including 92Y, 94S, 96A, 98L, 99A, and 100G), in which 91R, 95Y are functions of *α*- and *β*-interaction and 100G is at the end of helix E; (4) 107-115aa domain (including 107D, 108R, 111N, 112G, and 115E) includes the functional loci 108R, 115E, and 116T (function of the possible linker interaction) and 112G (bend between helices F′ and F); (5) 130-140aa domain, in which loci 130A, 133I, 135 K, 139V, and 140A are located at the eighth helix (124-143aa); (6) C-terminal 167-172aa domain in which loci 172V, 170D, 168Y, and 167S are functions of possible interaction and; (7) 164E involved trimer-trimer interaction of hexamer formation. There were only four loci (K28, I60, T75, and L161) of *β*-PE polypeptide sequences similar to HL-*Prochlorococcus* *β*-PE but different from LL-*Prochlorococcus*.

A fragment 66-74aa (LKNAGEAGD) in PEA of *K. alvarezii* was an extension in red algae compared with PEA in LL-*Prochlorococcus* (Figure 4(b)).

### 3.4. Conservation Domains of PC and APC

**Figure 5:**
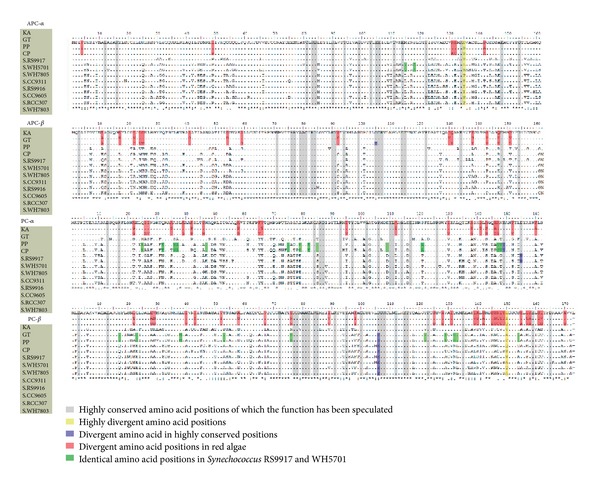
Highly conserved or divergent amino acid loci in APC and PC sequences of *Kappaphycus alvarezii*.

The *α*- and *β*-subunits of PC and APC in *K. alvarezii*, *G. tenuistipitata* (GT), *Porphyra purpurea* (PP), *C. paradoxa*, and* Synechococcus* (RS9917 (no PE), WH5701 (no PE), WH7805 (PEI), CC9311 (PEI, PEII), RS9916 (PEI, PEII), CC9605 (PEI, PEII), RCC307 (PEI, PEII), and WH7803 (PEI, PEII)) were chosen to analyze the conversion domains (Figure 5). The alignment of these sequences showed that the identity of *α*-subunit APC in *K. alvarezii* scored from 72.0 to 75.5% out of the other algae listed in this paper, *β*-subunit APC is from 78.3 to 82.7%, *α*-subunit PC is from 63.8 to 70.3%, and *β*-subunit PC is from 66.4 to 70.5%. The identities of *α*- and *β*-subunits APC between *K. alvarezii* and other algae are both higher than the identities of *α*- and *β*-subunits PC between *K. alvarezii* and other algae.

Judging from amino acid sequences in Figure 5, 80 conserved loci were shown, in which all reported that functional amino acids could be found. Loci 135 in *α*-subunit APC and 150 in *β*-subunit PC were highly divergent in all sequences aligned in this paper. In *α*-subunit APC, *β*-subunit APC, *α*-subunit of PC, and *β*-subunit of PC, there were 5, 15, 20, and 28 divergent loci with known functions between *K. alvarezii*, *G. tenuistipitata*, *P. purpurea*, and other algae. The identity between *β*-subunit PC in RS9917, WH5701 (without PE) and *K. alvarezii*, *G. tenuistipitata*, *P. purpurea*, and *C. paradoxa* was higher than other cyanobacteria, which were not found in *α*-subunit PC and *α*- and *β*-subunits APC. Moreover, there are 13 and 10 loci identical in *α*- and *β*-subunits PC of RS9917, WH5701 but different from other cyanobacteria, which are more divergent than *α*- and *β*-subunits APC. Most of these divergent subunits are similar to *K. alvarezii*, *G. tenuistipitata,* and *P. purpurea*, which are conserved in red alga, while 10 loci are specially unique only in both RS9917 and WH5701.

## 4. Discussion

Based on the data of PSII photochemistry in this study, *K. alvarezii* is a low-light-adapted species, but it has some regulative mechanisms for both excessive and deficient light. The *I*_*k*_ of *K. alvarezii* is less than 115 *μ*mol m^−2^ s^−1^, which is consistent with the results of chlorophyll fluorescent measurement in *Polyneura hilliae*, *Porphyra leucosticta*, and *Porphyra umbilicalis* [34–36]. This revealed that these red algae favor relatively weak light conditions. Yield II values of *K. alvarezii* demonstrated the variation of photosynthetic efficiency under different light intensities. Electron transport rate decreased under deficient or excessive light; however, the rate increased after four days of adaptation. Similar results were shown by RLCs. The results implied that *K. alvarezii* possessed both high- and low-light adaptability.

Variation of composition and gene sequences may reflect the evolution of PBPs in response to light [37]. When comparing PE amino acid sequences of* K. alvarezii* with two ecotypes of *Prochlorococcus *(Figure 4), it is shown that many loci in *K. alvarezii* PE sequences were conserved with LL-*Prochlorococcus*. However, loci conserved with HL-*Prochlorococcus* but divergent with LL-*Prochlorococcus* were also found. The divergent loci were mainly located in *α*-helix and had several reported functional domains (e.g., subunits' interaction, linkers' interaction, and chromophores' interaction). According to the previous studies [38], PE genes in LL-*Prochlorococcus* suffered from positive selection, and the selected loci were related to light-harvesting or energy-transferring. We speculate the mutative domains or loci were related to light responses of *K. alvarezii*. The extension domain near the chromophores' interaction area in PE-*α* of red algae, lacking in LL-*Prochlorococcus*, is also hypothesized to have an effect on low-light response. Further experiments are still needed to prove these assumptions. Moreover, by comparing APC and PC (Figure 5), we also found that APC sequences are more conserved than PC and *β*-subunits are more divergent than *α*-subunits in *K. alvarezii* and other algae. Although PBP sequences are divergent in all algae, we found that the loci with basic function are more conserved, such as subunits' interaction, linkers' interaction, and chromophores' interaction. The conservation may ensure PBPs' function in absorption and transfer of light and energy. The diversities of PE and PC may take part in algal surviving and reviving under different light conditions.

Our study evaluated photosynthetic characteristics of *K. alvarezii* and attempted to explain light adaptation by analyzing secondary and tertiary structure of photosynthetic protein. The results of this study would shed some light on understanding the potential mechanism of light response of *K. alvarezii *and other red algae.

## Figures and Tables

**Table 1:** Degenerate PCR primers of PBP genes.

Primers	Sequence	Direction	Target
KPE-1	5′-ATGCTTGAC(T)GCA(G)TTTTCT(C)AG-3′	Forward	CPE
KPE-2	5′-TTAGCA(G/T)TAA(G)A(T)GA(C)GTTGATG(T)ACG(A)-3′	Reverse	CPE
KPC-1	5′-ATGT(C)TAGAT(C)GCATTTGCC(T)AA-3′	Forward	CPC
KPC-2	5′-TTAG(A)CTTAGCGT(C)ATTAATAGC-3′	Reverse	CPC
KAPC-1	5′-ATGAGTATTG(A)TTACA(T/G)AAG(A)TC-3′	Forward	APC
KAPC-2	5′-TTAACT(C)TAA(G)A(G)CCAGAAC-3′	Reverse	APC

**Table 2:** Photosystem II efficiency of *K. alvarezii* ^1^.

Yield^2^	rETR	rETRmax	*I*_*k*_ (*μ*mol m^−2^ s^−1^)
0.516 ± 0.022	8.783 ± 1.379	24.606 ± 1.968	103.415 ± 10.258

^1^Plants are cultivated under the light intensity of 40 *μ*mol m^−2^ s^−1^.

^2^Indicated data is presented as the mean ± SD (*n* = 3).
